# Leveraging Large Language Models for Early Detection of Anomaly Work Injury Cases: Data-Driven Approach to Rehabilitation Efficiency

**DOI:** 10.2196/80607

**Published:** 2026-01-30

**Authors:** Peter Q Chen, Hayley Y W Gu, Heidi K Y Lo, Wing Chung Chang, Cameron J M Lai, Sun H S Lai, Andy S K Cheng, Peter H F Ng

**Affiliations:** 1 Department of Computing, Hong Kong Polytechnic University, PQ710, Mong Man Wai Building, Hong Kong, China (Hong Kong), 852 27667248; 2 Department of Psychiatry, University of Hong Kong, Hong Kong, China (Hong Kong); 3 Total Rehabilitation Management (HK) Limited, Hong Kong, China (Hong Kong); 4 Department of Health and Physical Education, The Education University of Hong Kong, Hong Kong, China (Hong Kong)

**Keywords:** anomaly detection, large language model, work injury, rehabilitation prediction, machine learning

## Abstract

**Background:**

Large language models (LLMs) have demonstrated potential in automating the analysis of unstructured clinical data, yet their application in rehabilitation therapy for work injury cases remains underexplored.

**Objective:**

We aimed to evaluate the performance of an LLM-assisted approach for the rapid identification of anomalous rehabilitation cases related to work injuries to enhance scalability and precision in case management.

**Methods:**

We retrospectively analyzed 110,346 deidentified work injury cases between 2001 and 2024 from a leading rehabilitation coordination company in Hong Kong, representing approximately 20% of all work injury incidents in the region. LLMs were used to estimate the expected duration of recovery based on free-text injury descriptions. The cases in which the actual number of medically certified sick leave days exceeded the LLM-predicted maximum were classified as anomalies.

**Results:**

The LLM-assisted method achieved high accuracy, with GPT-4o achieving over 73% accuracy in nonanomalous classification and 79% accuracy in all dataset detection, outperforming comparator models. The model maintained high accuracy across subgroups and demonstrated the reliable extraction of information from free-text notes.

**Conclusions:**

The proposed method demonstrated robustness when evaluated on a large-scale dataset with a bimodal age distribution. This study highlights the potential of LLMs to transform rehabilitation workflows by automating anomaly detection at scale. The method also shows promise in tailoring rehabilitation strategies to age-specific needs and leveraging LLM tools for efficient case management. However, a key limitation is that the dataset includes only injury cases from a single geographic region, potentially limiting the generalizability of the findings to other populations or health care systems.

## Introduction

Efficient and targeted rehabilitation management is essential to ensure that individuals with severe conditions receive timely and appropriate care [1]. However, current work injury management often lacks the precision needed to allocate resources optimally, resulting in delays and inefficiencies in addressing high-priority cases [2]. A core challenge lies in the misallocation of attention and services, where relatively minor injuries with predictable recovery trajectories are sometimes treated with the same urgency as more complex cases. This misdirection not only burdens the health care system but also diverts valuable clinical time and expertise away from anomalies, cases involving severe injuries or irregular patterns of recovery that require specialized evaluation or intervention [3]. Without a robust mechanism to distinguish these cases early in the workflow, rehabilitation systems risk compromised patient outcomes, inefficient use of limited resources, and waste critical resources. In Hong Kong, the term “anomalies” refers to cases where the injured worker’s records indicate a severe injury requiring special or intensive care [4]. These cases may also suggest potential discrepancies, such as claims that have been overstated for additional compensation or legal benefits, indicating that the incident may not follow typical recovery patterns according to industrial practice. In the Asia-Pacific context, including Singapore’s employment practices guidance and Australian public sector leave management, cases that exceed expected or allowable sick leave provisions are treated as requiring attention, which aligns with our operational use of sick leave exceedance to flag potential anomalies [5-7]. Nonanomalous data refer to cases in which the injured worker experiences a light injury expected to heal in the usual course, with a standard recovery process leading to a timely return to work. These records represent the standard outcomes, without complications or indications of potential fraud. Some anomalies may also signal potential inconsistencies, such as exaggerated claims made for extended compensation or legal advantage. In industrial practice, comprehensive annotations about why a case is “special” are typically unavailable. The only consistent, objective post hoc indicator of atypical recovery trajectory is the realized count of medically certified sick leave days. Consequently, this study operationalizes cases requiring attention via a fast filter that flags cases whose realized sick leave exceeds a large language model (LLM)−estimated expected range. We emphasize that this is a pragmatic triage proxy, not a clinical determination of pathophysiology or fraud.

Addressing this challenge requires innovative methods for quickly and accurately identifying severe cases to optimize the distribution of resources. With the advancement of artificial intelligence (AI) techniques, clinical decision support systems have been increasingly employed across various domains to assist therapists in decision-making [8-11]. In the context of workplace injury, recent research has integrated machine learning methodologies, such as the variational autoencoder, for predicting sick leave outcomes and establishing a high alertness cliff [12]. Nevertheless, the prediction process still partially depends on the initial judgment of work injury case managers, who serve as the primary decision-makers in these cases. Senior work injury case managers consistently achieve higher accuracy compared to AI-based predictions [13]. Even with the assistance of neural networks, AI cannot rapidly achieve an acceptable level of performance without proper data preprocessing and customization. The research gap lies in the lack of efficient, data-driven methods to proactively identify anomalies in work injury management workflows. Current practices predominantly rely on random case assignment and retrospective corrections, resulting in wasted time and resources.

Recently, LLMs have demonstrated exceptional capabilities in processing language-related data, even passing the United States Medical Licensing Examination [14,15]. It has also been studied in several medical fields [16-18]. Numerous studies and surveys have investigated LLMs’ ability to assume specific roles based on provided profiles, with results indicating that LLMs can effectively simulate profiled characters [19-21]. Simply prompting LLMs with a data description can generate responses in less than 1 minute without requiring additional model training. However, a critical research gap remains in determining how to constrain the outputs of LLMs and how to effectively design methods that leverage LLMs to detect anomalies in incoming injury cases efficiently.

Unlike traditional rehabilitation workflows, where senior work injury case managers must spend considerable time manually identifying anomalous cases, we developed an LLM-assisted method to streamline and accelerate this process. By leveraging prompt engineering techniques, we structured the input and constrained the output format to support accurate and efficient initial screening. The method is grounded in clinical reasoning: each injured worker is expected to follow a typical recovery trajectory, reaching a work-ready state within a medically anticipated time frame based on the nature and severity of the injury. LLMs, trained on extensive digital corpora that include medical and occupational content, are well positioned to infer such expectations and assist in detecting deviations from normative recovery patterns [22].

The scenario mirrors current practices in work injury management, where cases are often assigned randomly to junior or senior work injury case managers, only to discover later that certain cases would have benefited from senior-level expertise from the outset. This misallocation frequently results in delays and inefficient use of resources.

To this end, we proposed a novel approach leveraging LLMs to detect anomalies in occupational rehabilitation in the context of work injury management. Our method offered a potentially fast, scalable, and highly accurate solution for identifying severe cases based on data from work injury cases. Furthermore, this research collected over 110,000 work injury cases from a local company in Hong Kong, which handles nearly 20% of the total work injury cases yearly [23]. The data were used to validate our method, and several pilot studies were conducted for feasibility assessment, including model selection. Meanwhile, this research aims to uncover the key factors that characterize anomalies in this dataset, providing deeper insights into the decision-making process and facilitating a more informed allocation of resources. Our objectives are 2-fold: (1) developing a robust LLM-based method for anomaly detection in work injury management and (2) utilizing exploratory data analysis to uncover potential age and work-injury patterns in these anomaly cases.

## Methods

### LLM-Assisted Anomaly Fast Detection Method

This method uses a fast and reliable alertness cliff to classify cases more effectively. If the total number of medically certified sick leave days for an injured worker exceeds this cliff, the case is flagged as potentially anomalous and prioritized for review by senior-level personnel or a detailed evaluation. An LLM predicts the expected duration of sick leave for each case from injury and accident descriptions. By comparing realized sick leave with the model-predicted recovery days, cases exceeding the cliff are classified as anomalies, and those within the expected range are considered normal. To enhance precision, 3 aggregation rules are used to define the decision cliff, referred to as the cliff: the maximum, the average, and the median of 3 independent LLM−generated recovery estimates. A case whose realized sick leave exceeds this cliff is classified as an anomaly. To mitigate variability and improve reliability, the LLM is queried 3 times per case using the same prompt structure, and the final decision is derived from the aggregated predictions to produce a robust, data-driven anomaly detection process [24].

**Figure 1.**
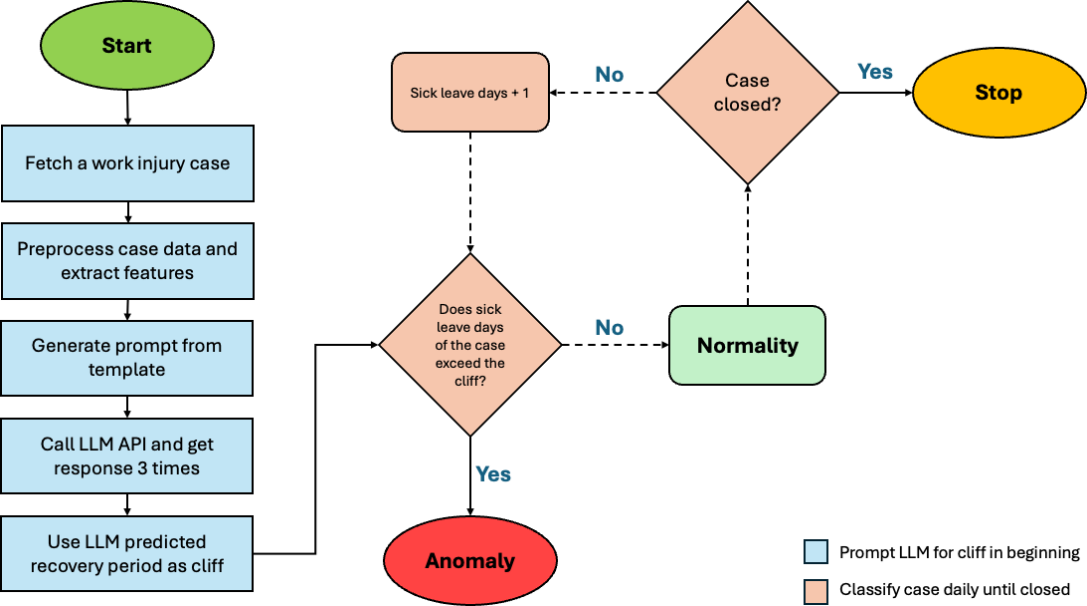
Large language model (LLM)−assisted anomaly fast detection method. API: application programming interface.

**Figure 2.**
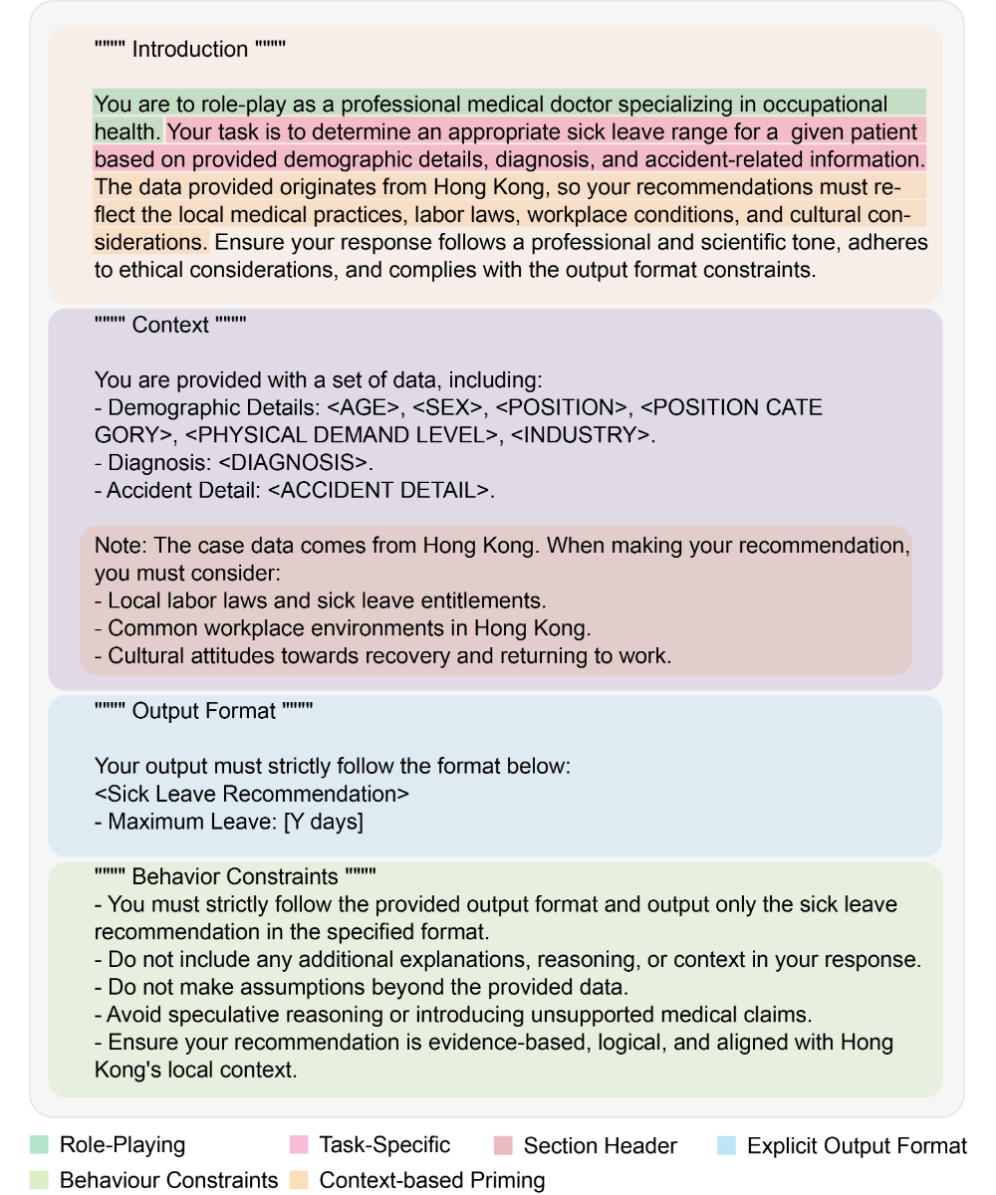
Concrete prompt used to acquire information from a large language model.

As illustrated in Figure 1, the workflow begins when a new work injury case is received. The “Query LLM 3 times and aggregate results” step rapidly determines the cliff using an LLM. The procedure begins by preparing case information, followed by preprocessing to retrieve demographics and extract key details, such as accident and diagnosis information. This content is embedded into a prompt based on the template shown in Figure 2. For each case, the LLM application programming interface (API) is queried 3 times to obtain predicted recovery periods, which serve as the cliff indicator for anomaly classification. A case is classified as nonanomalous if its sick leave has not yet exceeded the predicted cliff. For ongoing cases, sick leave days are incremented and reassessed against the cliff until the case is closed. Once the sick leave surpasses the cliff, the case is classified as an anomaly and referred to a senior work injury case manager for intervention.

The primary outcome is the accuracy of anomaly and nonanomalous classification in work injury cases, assessed on a dataset from a leading local work injury management company with expert-verified labels. The systematic use of LLM predictions enhances detection sensitivity while reducing manual workload, enabling case managers to focus on genuinely complex cases that require expert attention.

In routine operations, rich clinical detail is often unavailable at intake, so triage relies on minimal text and basic demographics. To enable low-latency and low-cost prioritization, a case-specific cliff is defined as the LLM-estimated expected duration of sick leave, serving as a data-driven prior on typical recovery given available notes. As the case progresses, if the running tally of medically certified sick leave exceeds this prior, particularly early in the timeline, the case is automatically queued for senior review. This mechanism functions as a workload triage heuristic rather than a diagnostic judgment, triggering timely escalation in cases of information scarcity, improving allocation precision, and deferring definitive clinical determinations to expert assessment.

The method was validated using a dataset of 110,346 real-world work injury cases provided by a leading work injury management company in Hong Kong.

### Prompt Template

Figure 2 demonstrates the detailed prompt engineering template, which utilizes multiple prompting techniques to enhance LLM performance. For clarity, the section header technique, such as “Introduction,” “Context,” “Output Format,” and “Behavior Constraints,” is used in the prompt template [25]. In the first part of the prompt template, the role-playing technique enhances contextual understanding, adaptability, and response accuracy by simulating specific personas, perspectives, or expertise in a given scenario [26]. To improve response relevance and coherence, the template specifies that all cases occurring in Hong Kong should utilize the context-based priming technique [27]. In the “Context” section of the prompt template, the input data for the injury case, including demographic details (eg, age, occupation), diagnosis details (textual description), and accident details (textual description), were included. In the “Output Format” part, the Explicit Output Format avoids undesired reasoning steps (eg, Chain-of-Thought) or other deviations, ensuring the LLM generates responses strictly in the intended structure without adding irrelevant content [28]. The “Behavior Constraints” part also serves a similar purpose, ensuring that the model’s responses remain factual, precise, and contextually appropriate. Prompts are explicitly contextualized to Hong Kong’s legal and clinical environment to enhance ecological validity, with strict output schemas for parsability and consistency [29]. Explicit instructional constraints are embedded to reduce hallucination and enforce adherence to the analytical task.

### Pilot Study

In our pilot study, we examined the varying strengths of different LLMs (eg, mathematical reasoning) and recognized that model size and architecture significantly influence performance [25,30-32]. To ensure a robust evaluation, we selected the largest and most widely recognized models from diverse LLM families, encompassing a broad range of architectures and capabilities.

Our primary objective was to determine whether these models could interpret a predefined prompt template and generate outputs that conformed to the required structure. Rather than examining their reasoning or predictive abilities, we focused on consistency, introducing 2 metrics: Compliance, which measures adherence to guidelines for producing the desired content, and Self-Consistency, which assesses whether the same response is generated across 3 repeated trials. We randomly sampled 100 cases from the dataset, prompted each model 3 times per case, and recorded the number of outputs that followed the required format. We focus on per-case anomaly triage using an upper bound on expected sick leave, so the prior work’s center-focused reproducibility framework and large-run benchmarking do not fit our objective, evaluation needs, or deployable low-stochasticity protocol [32].

**Figure 3.**
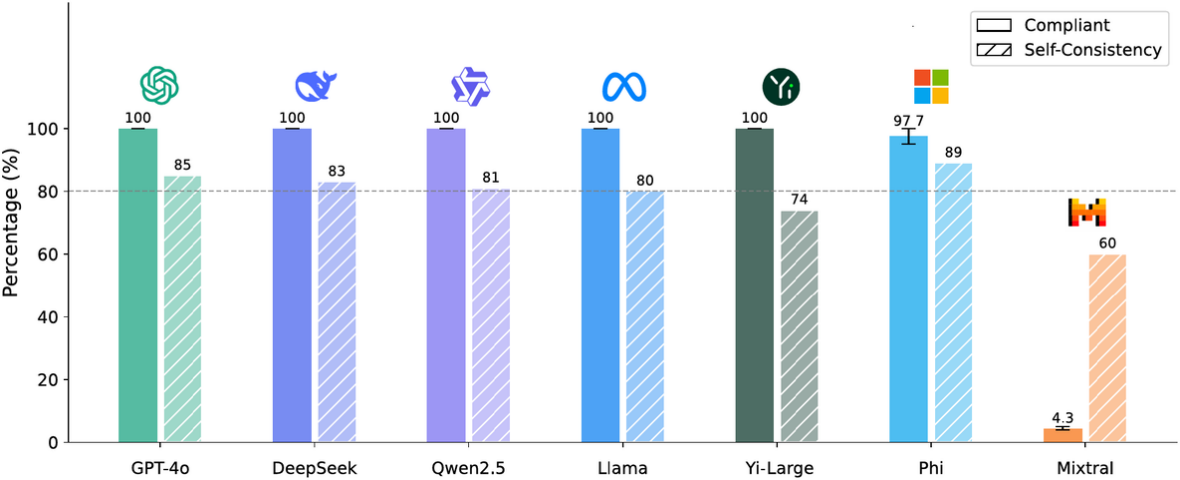
Compliance and self-consistency of different large language models.

Figure 3 presents the results of this pilot study. Compliance represents the proportion of responses that satisfied our prescribed guidelines, while Self-Consistency quantifies each model’s consistency across repeated prompts. Among 7 leading commercial LLMs, ChatGPT-4o, DeepSeek-V3, Qwen2.5-72B Instruct, Llama-3.1-405B Instruct, and Yi-Large achieved perfect Compliance; other models failed to avoid generating undesired content. We also examined internal consistency by comparing outputs across 3 prompts, categorizing them as identical across all trials, identical in at least 2, or unique each time. ChatGPT-4o exhibited the highest Self-Consistency, and several other models met our chosen 80% cliff for the subsequent experiments.

However, certain models with high Self-Consistency struggled with Compliance. This discrepancy is especially concerning, given our objective of providing a fast and reliable anomaly detection system for work injury management companies. Strict adherence to instructions is critical: any deviation can introduce erroneous or fabricated data, ultimately undermining the detection process. Ensuring compliance is thus essential to maintain the integrity of anomaly detection.

### Work Pipeline

Based on the results of the pilot study, we selected DeepSeek-V3, ChatGPT-4o, LLaMA-3.1-405B-Instruct, and Qwen2.5-72B to serve as LLM agents in our framework. All selected models achieved 100% compliance with the prompt template and demonstrated over 80% self-consistency across repeated responses.

A request-response framework was implemented using the FireWorks API in Python. Decoding parameters were configured for low-variance outputs using a temperature of 0.2 and a top-p of 1.0, which empirically reduced variability while maintaining robust adherence to the required output schema. A temperature of 0.0 was considered for full determinism; however, some models exhibited occasional output truncation or schema noncompliance at strictly deterministic settings during preliminary checks.

The demographic information, incident accident details, and clinical diagnoses were embedded into a standardized prompt template, as shown in Figure 2. Each prompt was submitted to the LLM via an API. A Python script extracted the responses to generate a predicted maximum duration of medically certified sick leave for each case. The data were then visualized to assess the accuracy of the LLM-based method.

### Data Sources

Our dataset originates from a leading work injury management company in Hong Kong, which manages approximately 20% of work injury cases annually, covering records from 2001 to 2024. The dataset comprises 110,346 cases, with a gender distribution of 41.3% female and 53.6% male. The study population is predominantly Chinese, with individuals ranging in age from 18 to 83 years. This broad demographic coverage provides a robust basis for analyzing patterns across different age groups and genders within a relatively homogeneous ethnic context. Input leakage was not possible in this study. All records are confidential medical data, fully classified, and never publicly released or exposed to the models beyond the controlled evaluation pipeline, thereby preventing any external contamination that could bias LLM outputs or compromise validity.

Within this dataset, 15,575 cases were recorded as having zero sick leave days, which were treated as potential data entry errors. We exclude zero-day legitimate cases as outliers because they are immediately escalated to senior rehabilitation coordinators on day zero and handled outside our fast detection system, which targets anomalies only after predicted sick leave durations are exceeded. An additional 9230 cases had nonzero sick leave durations but contained missing values. For the primary predictive analysis, we used 85,541 cases that reported a nonzero number of medically certified sick leave days and had no missing data. These were inputs for the LLMs to predict the expected duration of normal recovery. Although excluded from the prediction task, the remaining data groups were also analyzed to extract relevant insights, given their substantial size.

### Data Preprocessing

The dataset underwent staged preprocessing to ensure consistency and analytical suitability. Records outside the target time window were removed, implausible values were constrained within reasonable bounds, and entries with nulls in critical analytical fields were excluded. Noncritical descriptive fields with missing values were imputed using a neutral placeholder to preserve coverage while signaling incompleteness.

Categorical features were standardized through controlled vocabulary mapping, consolidation of multivalued entries into explicit multicategory indicators, and aggregation of low-frequency categories to mitigate sparsity. Text fields were sanitized by removing noninformative placeholders, and duplicates were eliminated based on content equivalence. A focused set of salient variables was retained for downstream analysis.

### Data Analysis

In this study, we utilize a comprehensive set of metrics to rigorously evaluate the performance of the LLM-assisted anomaly detection method, encompassing classification accuracy, error magnitude, and model reliability. Classification accuracy, a core metric, is calculated based on 3 cliff-based methods: method 1 (maximum of 3 LLM predictions), method 2 (average of 3 predictions), and method 3 (median of 3 predictions). To further assess prediction deviations between realized sick leave days and LLM-predicted cliffs, we compute mean absolute error (MAE), mean squared error, root mean squared error (RMSE), mean absolute percentage error (MAPE), and mean percentage error (MPE). Additionally, Compliance (adherence to structured output formats) and Self-Consistency (reproducibility of outputs across repeated prompts) are quantified to ensure model reliability. We evaluate misclassification deviation, summarized through percentiles (eg, 50th and 75th percentiles), to analyze error distribution. Furthermore, exploratory data analysis is conducted to provide insights into the dataset, including descriptive statistics such as injury frequency, demographic distributions (eg, age, gender, occupation), anomaly prevalence, and misclassification patterns across key variables like body part and industry type. Together, these metrics and calculations form a robust framework for assessing the precision, reliability, and operational effectiveness of the proposed LLM-based anomaly detection system.

We evaluated triage performance using cliff-based classification derived from LLM-predicted “cliffs” of expected sick leave duration. For each case, the LLM was queried 3 times with the same prompt. The per-case decision cliff was then defined by 1 of the 3 aggregation rules: method 1, method 2, and method 3. A case was classified as an anomaly if it realized medically certified sick leave days exceeded the chosen cliff; otherwise, it was classified as nonanomalous. The primary performance metric was accuracy, computed as the proportion of correctly classified cases over the evaluation set, and reported overall and stratified by anomaly and nonanomalous subsets to characterize trade-offs across decision rules and models.

To assess reliability and error magnitude, we further quantified misclassification deviation, defined for errors as the absolute difference between realized sick leave days and the decision cliff, summarized via percentiles (eg, 50th and 75th).

To gain a deeper understanding of the phenomenon in the dataset, salary is treated as a composite proxy that captures differences across job types, seniority, contract structures, and work experience. Given the absence of granular role-level pay scales, salary should not be interpreted as a pure measure of experience but as an indicator shaped by occupational category and tenure.

### Ethical Considerations

This study introduced an innovative anomaly detection method for work injury rehabilitation, validated using real-world cases from Hong Kong. The project has been approved by the PolyU Institutional Review Board (reference HSEARS20250406002). A pilot study was first conducted to evaluate the performance of several well-known commercial LLMs, which informed the selection of the most effective models for the subsequent experiments.

The dataset was provided by one of the largest work injury management companies in Hong Kong, which manages nearly 20% of the government-reported work injury rehabilitation cases. The data were shared exclusively for research purposes under a strict confidentiality agreement. Prior to transfer, all records were anonymized by the provider to ensure the protection of personal information.

## Results

### Work Injury Dataset

**Figure 4.**
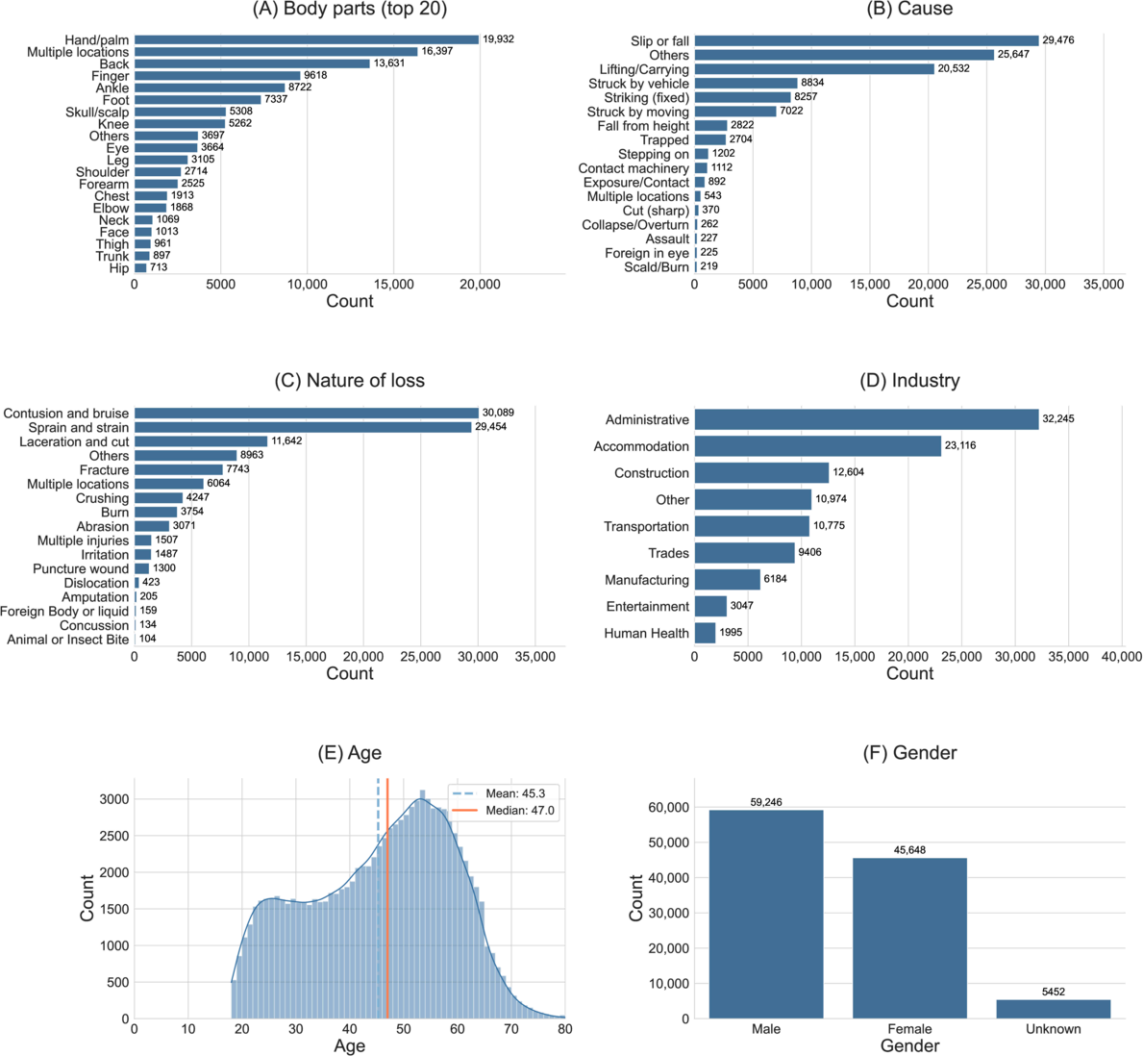
Demographic data statistics in the dataset. (A) The top 20 most common categories of injured body parts. (B) Most common categories in the industry. (C) Most common categories in the nature of loss. (D) Most common categories in the cause of injury. (E) Gender distribution in the dataset. (F) Age distribution in the dataset*.*

Figure 4 presents a comprehensive analysis of 110,346 deidentified occupational injury cases managed by a leading rehabilitation coordination company in Hong Kong between 2001 and 2024. Injuries predominantly involved peripheral anatomical regions, with fingers (n=16,397) and backs (n=13,631) collectively accounting for nearly one-quarter of all incidents, followed by hand or palm injuries (n=9618) and ankle injuries (n=8722). Consistent with these anatomical findings, the most common types of loss were contusions or bruises (n=30,089) and sprains or strains (n=29,454), whereas open wounds, such as lacerations and cuts (n=11,642), and fractures (n=7743), occurred less frequently. Industry-specific data indicate a substantial burden arising from labor-intensive service sectors, notably “Administration and support services” (n=32,245) and “Accommodation and food service activities” (n=23,116), collectively accounting for more than half of all cases and surpassing the construction sector (n=12,604) in this dataset. Precipitating events were predominantly same-level slips, trips, and falls (n=29,476) and manual lifting or carrying tasks (n=20,532). Demographically, male workers represented a modest majority (53% of claims); nevertheless, female workers comprised a substantial proportion (42%). The age distribution was right-skewed, with a mean age of 45.3 (SD 13.3) years and a median of 47.0 (IQR 21.0) years.

### Performance Assessment of LLMs

Table 1 shows the LLM classification accuracy across the selected models for different data categories. All LLMs mentioned in Figure 3 have been tested. For the entire dataset, all selected LLMs achieved more than 70% accuracy in the maximax, expected maximum, and median cliff criteria methods. Among the selected LLMs, GPT-4o achieved the best performance in all cliff criteria, while DeepSeek-V3 had the lowest performance. In the separate anomaly dataset containing only anomalies, the prediction accuracy of all selected models exceeds 95%. The best performance for the nonanomalous dataset comes from GPT-4o as well, achieving more than 76% under maximax cliff criteria, over 73% accuracy under expected maximum cliff criteria, and a median cliff criterion.

**Table 1.** Case classification accuracy across models^a^.

	Method 1 (%)	Method 2 (%)	Method 3 (%)
Qwen	78.71	78.26	78.19
DeepSeek	76.31	75.75	75.66
Llama	78.15	76.93	76.76
GPT-4o	81.77	79.95	79.51
Anomaly			
Qwen	97.72	97.79	97.79
DeepSeek	97.59	97.81	97.80
Llama	97.69	98.02	97.97
GPT-4o	96.32	97.16	97.21
Nonanomalous			
Qwen	71.71	71.07	70.98
DeepSeek	68.48	67.62	67.51
Llama	70.96	69.16	68.95
GPT-4o	76.40	73.61	72.99

a Method 1 uses the maximum value among the 3 large language models (LLMs) predictions as a cliff to classify anomalies and nonanomalous. Method 2 uses the average of the 3 LLM predictions as a cliff for classification. Method 3 uses the median of the 3 LLM predictions as a cliff for classification.

Table 1 further highlights the trade-offs in classification accuracy when different criteria are applied. When using the expected maximum as the classification criterion, the model achieves higher accuracy in anomaly detection but at the cost of reduced accuracy in nonanomalous classification. However, misclassifying nonanomalous cases is relatively less consequential, as such cases typically resolve quickly, with injured workers returning to work in a short period. In contrast, misclassifying anomalies can have significant financial and operational implications. Suppose an anomaly is incorrectly classified as a normal case. In that case, the company may need to allocate additional resources to reassign a senior work injury case manager later in the process, leading to prolonged recovery times and potentially missed rehabilitation windows. From an anomaly detection perspective, Llama demonstrates the highest detection rate. However, when considering overall performance across both anomaly and nonanomalous classification, GPT-4o outperforms other models, making it the most balanced and practical choice for real-world deployment. These findings highlight the importance of selecting an LLM that optimally balances accuracy, adherence to instruction, and overall classification performance.

Table 2 shows that across the 3 aggregation strategies, absolute and squared errors remain high: MAE is approximately 72 days, and RMSE is around 158 days for all methods, indicating substantial pointwise deviations and volatility in predicting the maximum sick leave duration. Relative errors are also large: MAPE ranges from 169.86% to 195.48%, and MPE exceeds 100% for all methods, evidencing pronounced systematic overestimation. Among the alternatives, the median-based cliff (method 3) yields the lowest relative error (MAPE=169.86%, MPE=106.77%) and slightly lower dispersion, whereas using the maximum prediction as the cliff (method 1) amplifies both bias and variance; the mean (method 2) lies in between. Despite these differences, the small gaps in MAE or RMSE across methods suggest that aggregation choice alone does not resolve the core error magnitude, and bias calibration or robustness enhancements are warranted. Directly using LLMs to predict sick leave duration from demographics yields large absolute and relative errors (≈72-day MAE, ≈158-day RMSE, MAPE >169% with systematic overestimation), revealing unstable and biased point forecasts, which motivates our shift to a fast exceedance−based detection method rather than relying on raw predictions.

**Table 2.** Standard metrics for 3 methods^a^.

	MAE^b^	MSE^c^	RMSE^d^	MAPE^e^ (%)	MPE^f^ (%)
Method 1	72.37	24,741.36	157.29	195.48	136.39
Method 2	72.42	25,173.20	158.66	172.39	110.32
Method 3	72.54	25,266.41	158.95	169.86	106.77

a Method 1 uses the maximum value among the 3 large language models (LLMs) predictions as a cliff to classify anomalies and nonanomalous. Method 2 uses the average of the 3 LLM predictions as a cliff for classification. Method 3 uses the median of the 3 LLM predictions as a cliff for classification.

b MAE: mean absolute error.

c MSE: mean squared error.

d RMSE: root mean squared error.

e MAPE: mean absolute percentage error.

f MPE: mean percentage error.

### LLM Misclassifications Case Study

**Figure 5.**
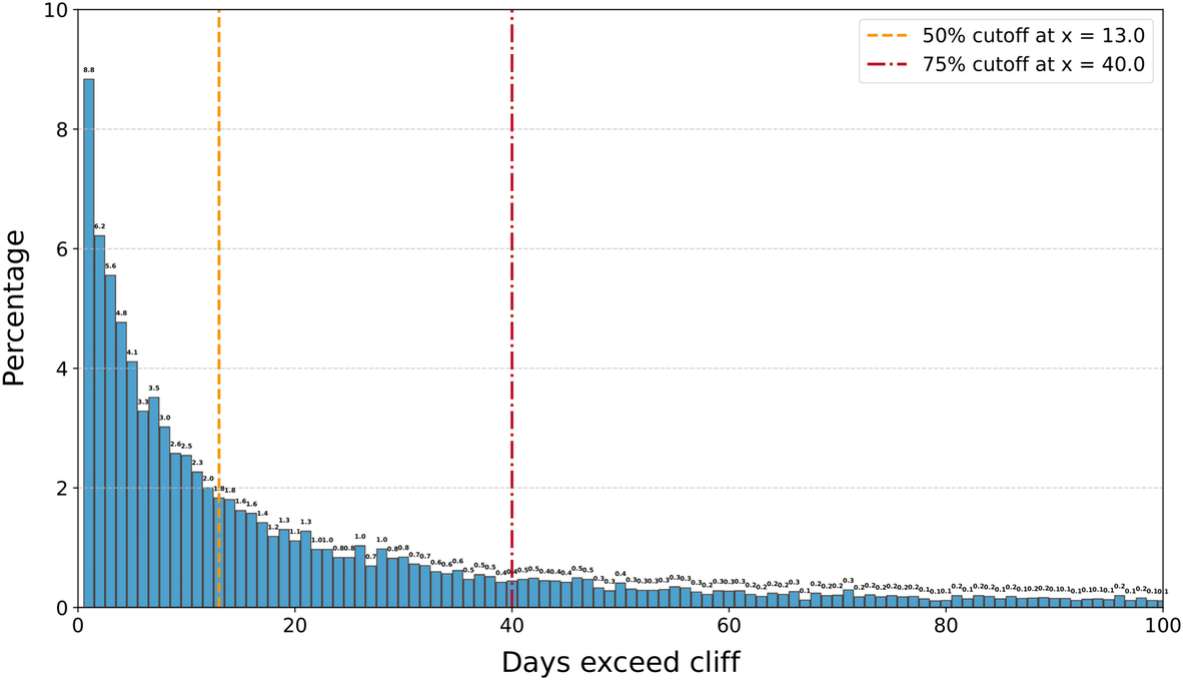
Distribution of erroneous predictions beyond the cliff.

Since method 1 achieved the highest overall accuracy, this section primarily focuses on analyzing the predictions of the LLM based on method 1, as well as those generated by ChatGPT-4o, which demonstrated the highest average accuracy among all the selected models. Figure 5 illustrates the distribution of erroneous predictions that exceed the specified cliff when method 1 is applied. The yellow dashed line represents the 50th percentile of the cumulative distribution, corresponding to a deviation of 13 days. In contrast, the 75th percentile of the cumulative distribution corresponds to a deviation of 40 days. These results indicate that half of the erroneous predictions deviate from the ground truth by no more than 13 days, further underscoring the robustness of the proposed method. The previous work on similar datasets shows that the traditional variational autoencoder could only achieve an average error of 107.447 days, and they failed to predict a cliff for classifying the anomaly and nonanomalous cases [12,13]. Table 2 shows the absolute errors of direct LLM predictions, demonstrating substantial bias, while Figure 5 highlights erroneous cases flagged by our fast detection method, underscoring that our approach is intended to assist rather than replace rehabilitation coordinators.

**Figure 6.**
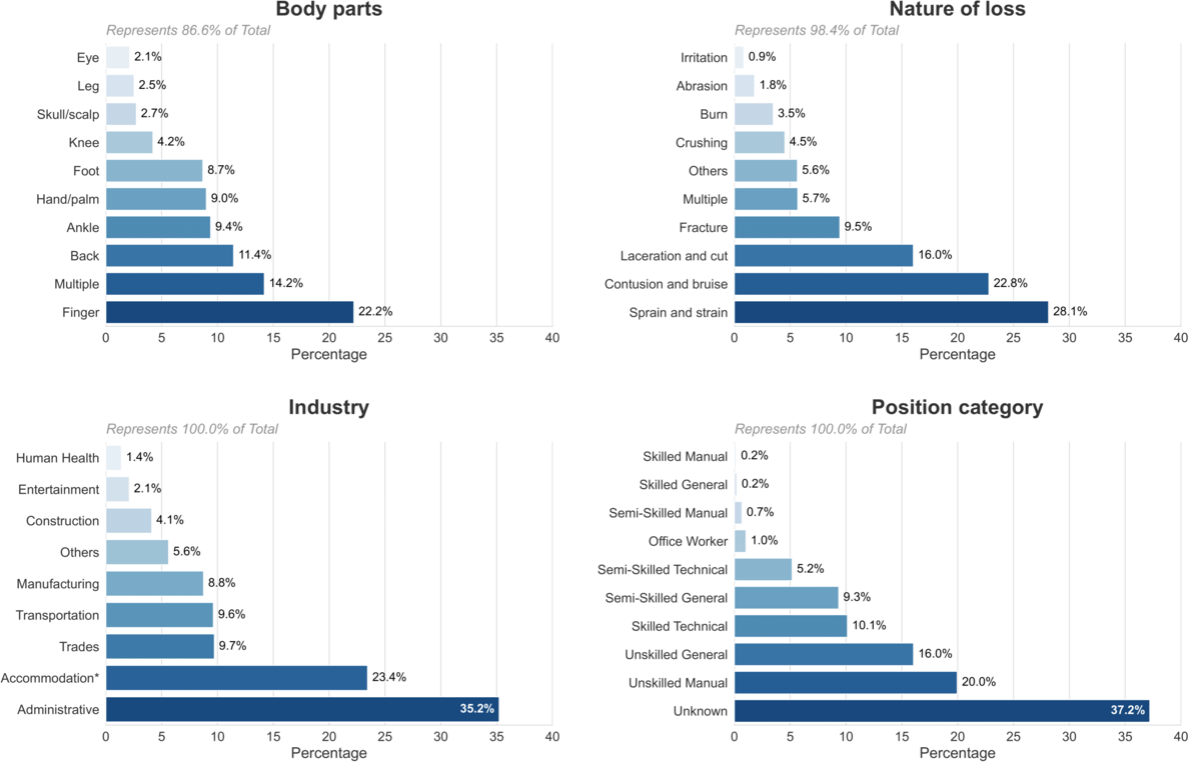
Distribution of large language model misclassifications nonanomalous across the key variables (top 10).

To gain a deeper insight into the LLM’s prediction performance, we examined the distribution of GPT-4o outputs using method 1 (as described in the previous section). Specifically, 14,751 nonanomalous cases were misclassified as anomalies, and 847 anomalies were misclassified as normal. Figure 6 illustrates the distribution of the key variables within these misclassified nonanomalous cases. Finger has the highest proportion of misclassifications, at approximately 23%, whereas other body parts each account for around 10%. In the “Nature of Loss” variable, “Sprain & Strain” accounts for over 28% of the misclassifications, followed by “Contusion & Bruise” and “Laceration & Cut,” both of which exceed 15%; the remaining categories each fall below 10%. Regarding the Industry, the Administrative and Support Service sector and the Accommodation and Food Service Activities sector exhibit notably higher proportions of misclassifications (over 20%) relative to others. Finally, in the “Position Category” variable, most misclassifications occur under the “unknown” category.

Figure 6 also suggests that certain misclassifications may arise from incomplete data, such as the absence of a position category, which impairs the LLM’s ability to predict the cliff accurately. Without this critical information, the model must rely on its intrinsic knowledge, leading to increased variability and uncertainty. Additionally, for variables such as the nature of loss, injured body parts, and industry type, human judgment is also prone to significant bias, particularly in those variables with high percentages of misclassification [13].

### LLM Direct Prediction Error Distribution

**Figure 7.**
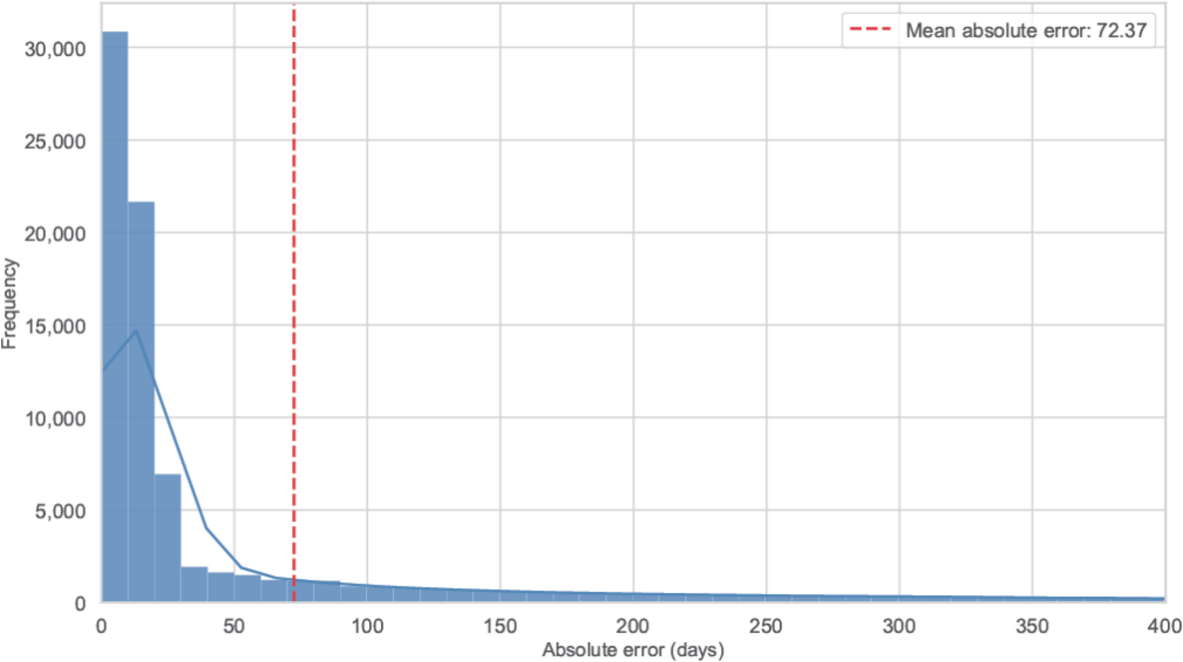
Distribution of the large language model raw prediction absolute error.

Figure 7 shows the distribution of absolute errors for raw LLM day predictions; the mean error is 72 days, which indicates that direct prediction yields deviations too large for practical use. Therefore, we use the LLM as a fast anomaly filter that flags cases whose realized sick leave exceeds an estimated upper bound, rather than predicting sick leave days directly.

### Case Study of Our Approach

In this case study, we analyze a 51-year-old female worker who sustained a back injury while lifting a heavy basket of packaged bread. The injury was classified under Body Parts: Back, Nature of Loss: Sprain & Strain, and Cause: Injured whilst lifting or carrying, with the worker employed as a packer in the Accommodation and Food Service Activities industry. The actual sick leave duration was 6 days, while the LLM predictions for recovery duration were consistent across all methods: result 1=14 days, result 2=14 days, and result 3=14 days, leading to cliff values of max_max=14 days.

The difference between the actual sick leave and the maximum predicted cliff sick leave duration max_max was –8 days, indicating that the actual recovery period was well within the predicted range and did not exceed the anomaly cliff. This demonstrates the effectiveness of the LLM-assisted method in identifying cases that conform to expected recovery patterns, ensuring that resources are not misallocated to cases that align closely with typical recovery trajectories.

### Exploratory Insights From the Dataset

**Figure 8.**
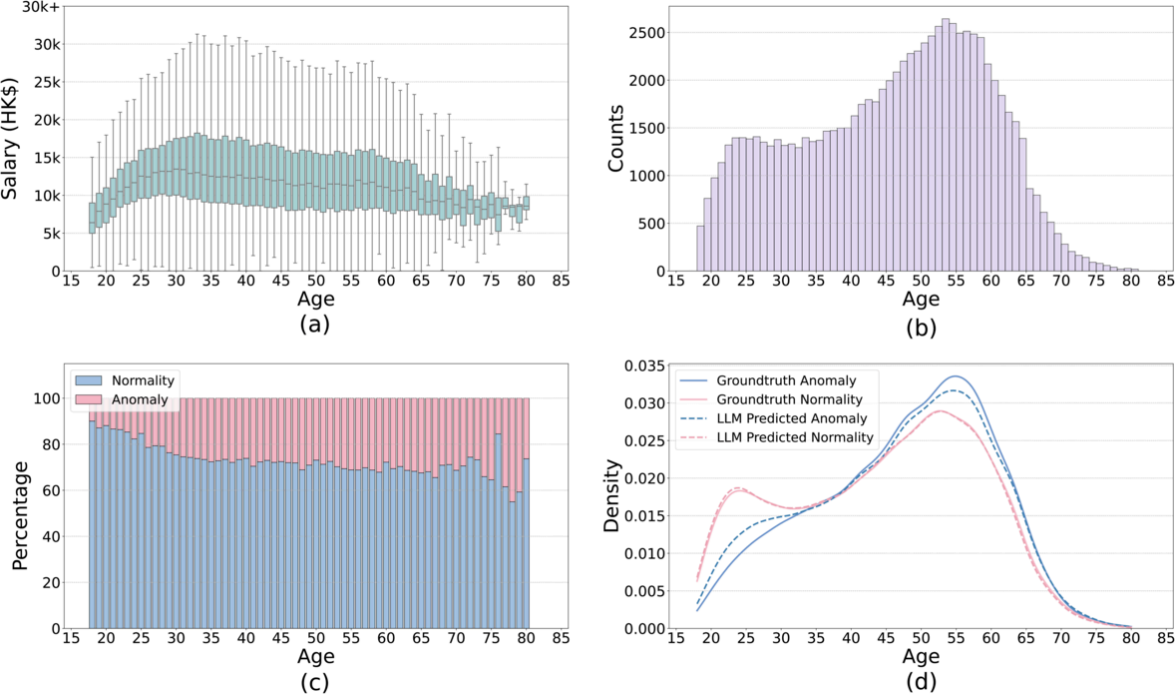
Overview of salary and age distributions with anomaly analysis. (A) Salary boxplot across ages. (B) Age distribution of all data. (C) Percentage of anomaly across ages. (D) Age distribution of anomaly or normality data.

To examine anomalies, we present a salary box plot against age in Figure 8. The top-left panel of the figure presents a box plot of salary against age, where the median salary for each age group is extracted to reveal a clearer trend. The median salary initially increases, peaking between ages 35 and 40, before gradually declining. The top-right panel illustrates the distribution of age counts from 18 to 80 years, showing an initial peak in the early 20s, followed by a decline until the 40s, after which the number of cases rises again, reaching its highest peak around the age of 55 years. The bottom-left panel displays the percentage of anomalies across different age groups, revealing a steady increase in anomaly prevalence with age. Finally, the bottom-right panel illustrates the normalized age distributions of both anomalies and nonanomalous, alongside the LLM’s predicted anomalies and nonanomalous. The red line (representing nonanomalous) closely mirrors the overall age distribution, while the blue line (representing anomalies) steadily increases and peaks near the age of 60 years. Notably, the LLM’s predicted distributions align closely with the ground-truth trends.

## Discussion

### Principal Findings

The performance of the proposed LLM-assisted anomaly fast detection method demonstrates promising results, 110,346 deidentified work-injury cases between 2001 and 2024 from a leading work injury management company in Hong Kong, representing approximately 20% of all work injury incidents in the region. Compared to previous research, which primarily assessed AI prediction accuracy by comparing it to work injury case managers’ judgments, our study provides a more comprehensive evaluation [12]. Prior studies demonstrated that AI predictions could surpass human work injury case managers in both nonanomalous and anomalous cases. However, while they also attempted to predict anomaly cliffs, their findings did not explicitly report the accuracy of such predictions. In contrast, our approach ensures that the accuracy of anomaly cliff prediction is systematically analyzed, contributing to a more reliable anomaly detection framework.

In Figure 7, we illustrate the trend of salary changes, considering salary as a key indicator of work experience. It is well known that physical ability peaks in the 20s, remains stable or slightly declines until around 30‐35, and then drops significantly thereafter [33,34]. Comparing this physical ability trend with the salary trend, we observe that workers in their early 20s possess peak physical strength but lack experience. As a result, they may be more prone to injuries; however, their quick recovery often prevents these cases from becoming anomalies. This explains the initial peak in injury cases around the age of 20 years.

By the time workers reach their 30s, they have gained significant experience (as indicated by higher salaries), while their physical ability has not yet declined substantially. Consequently, the number of work injury cases decreases between the ages of 30 and 40 years. However, after 40, salaries may begin to decline slightly as workers are unable to maintain the same working hours as before, while their physical ability drops sharply. This results in a second peak in work-related injury cases. At this stage, injuries are more severe, and recovery is less likely, contributing to an increase in both the number and proportion of anomalies.

### Limitations and Future Directions

This study tests LLMs for spotting unusual rehabilitation cases. The models read injury diagnoses and accident notes, estimate a normal recovery time, and flag cases that fall outside that range. We judge the approach by how well it separates nonanomalous from anomalies, not by exact prediction accuracy. However, the limitations are that no comparison is made with human rehab coordinators, only general-purpose LLMs without medical models, and data from a single region; therefore, the findings may not be generalizable. As LLMs improve, they could streamline the rehabilitation triangle and resource planning. This research can be readily transferred to other rehabilitation systems within Hong Kong, but adaptation to other regions would be domain specific due to significant differences in work injury frameworks, legal and policy requirements, and cultural practices.

Deterministic decoding with temperature 0.0 could further enhance reproducibility. As a robustness check, future or supplementary analyses can compare performance and schema compliance at 0.0 versus 0.2 to quantify any trade-off between determinism and adherence to output constraints. If comparable, deployment would favor 0.0 to maximize reproducibility; if not, a small nonzero temperature remains justified to preserve formatting reliability under operational constraints.

### Conclusions

We present an LLM-based approach that estimates expected recovery time from injury records and flags deviations as anomalies, streamlining rehabilitation triage. Tested on more than 110,000 Hong Kong work-injury cases, the method improved classification efficiency; GPT-4o delivered the most balanced accuracy, with DeepSeek-V3 and Qwen2·5-72B Instruct close behind. Demographic analysis reveals that injuries are more frequent yet milder in younger workers, whereas those aged 40 and above experience more anomalies, reflecting reduced resilience. The approach advances data-driven rehabilitation coordination and optimizes resource allocation.

## Abbreviations

AI: artificial intelligence
API: application programming interface
LLM: large language model
MAE: mean absolute error
MAPE: mean absolute percentage error
MPE: mean percentage error
RMSE: root mean squared error
